# Management of cetuximab-induced skin toxicity with the prophylactic use of topical vitamin K1 cream

**DOI:** 10.2478/v10019-010-0036-6

**Published:** 2010-09-22

**Authors:** Janja Ocvirk

**Affiliations:** Institute of Oncology Ljubljana, Ljubljana, Slovenia

Cetuximab is an immunoglobulin G1 monoclonal antibody that binds to the extracellular domain of the epidermal growth factor receptor (EGFR) blocking ligand-induced auto-phosphorylation and subsequent receptor mediated signalling.^1^,^2^ Cetuximab in combination with chemotherapy is effective in the treatment of EGFR-expressing tumors including metastatic colorectal cancer (mCRC).^2^,^3^

EGFR is strongly expressed in the keratinocytes, cells of eccrine and sebacceous glands and in the epithelium of hair follicles, and is important for normal skin development and function.^4^ Blocking cutaneous EGFR signalling with EGFR inhibitors leads to a spectrum of skin reactions which occur in ≥80% of patients, the most common being acneiform rash which occurs most frequently on the head and neck regions and on the trunk. Other less frequent reactions include, pruritus, dry skin, desquamation, hypertrichosis, and paronychia.^1^,^2^ Approximately 15% of cutaneous reactions are severe (≥ grade 3; US National Cancer Institute–Common Toxicity Criteria)^5^, causing cetuximab therapy to be interrupted.^6^

We have investigated the prophylactic treatment of patients with a topically applied skin cream containing urea and 0.1% vitamin K1 (Renconval K1^®^) during cetuximab therapy. The aim of the study was to continue cetuximab without treatment delays or dose reductions, which may impact on tumour response rates.^7^ Four patients with mCRC receiving first-line cetuximab in combination with chemotherapy, had applied vitamin K1 cream facially twice daily for 8 weeks from the first infusion of cetuximab. Patients were screened weekly and photographs taken. The study was performed in accordance with the Declaration of Helsinki (5^th^ revision, October 2000) of the World Medical Association^8^ and approved by the National Medical Ethics Committee of the Republic of Slovenia. Patients provided written informed consent.

During treatment, no topical or oral antibiotics were prescribed and other moisturizers were not needed. Only one patient was judged to have developed mild facial papules and all four patients developed acneiform eruptions on the trunk ranging from mild to severe. The grade of acneiform rash was reduced where vitamin K1 cream was applied as prophylaxis (Table 1 and Figure 1).

At the end of cetuximab treatment one complete response, one stable disease and two partial responses were recorded.

Vitamin K activates EGFR signalling; preclinical studies have shown that 0.1–0.5 mM vitamin K3 completely abrogated EGFR inhibition *in vitro* and was associated with upregulation of phosphorylated EGFR in the skin when used in topically applied cream.^9^,^10^ In a study of 30 patients treated with Reconval K1^®^ on the first appearance of acneiform rash, we previously reported a median time to improvement of 8 days, and down-staging of rash by ≥1 grade after 18 days. No cetuximab dose reductions or treatment delays were required in patients with grade ≤2 cutaneous toxicity and no toxicities associated with Reconval K1^®^ were reported.^7^,^11^

In the present study we investigated the prophylactic use of vitamin K1 cream to the face in comparison with the trunk, which received no treatment. Whilst curative treatment has already been reported to be effective^7^, prophylactic treatment is potentially more effective. No cetuximab dose reductions or treatment delays were required. The topical use of vitamin K1 cream for preventing or reducing cetuximab-related acneiform rash appears to be promising.

It remains very important to treat skin reactions related to EGFR inhibitors promptly to ensure a better patient quality of life without dose reduction or drug discontinuation. We conclude that Reconval K1^®^ has potential for prophylactic use in the treatment of cetuximab-related skin toxicity, but that further studies are required to evaluate the impact of its use on tumor response rates and patient quality of life.

## Figures and Tables

**FIGURE 1.**
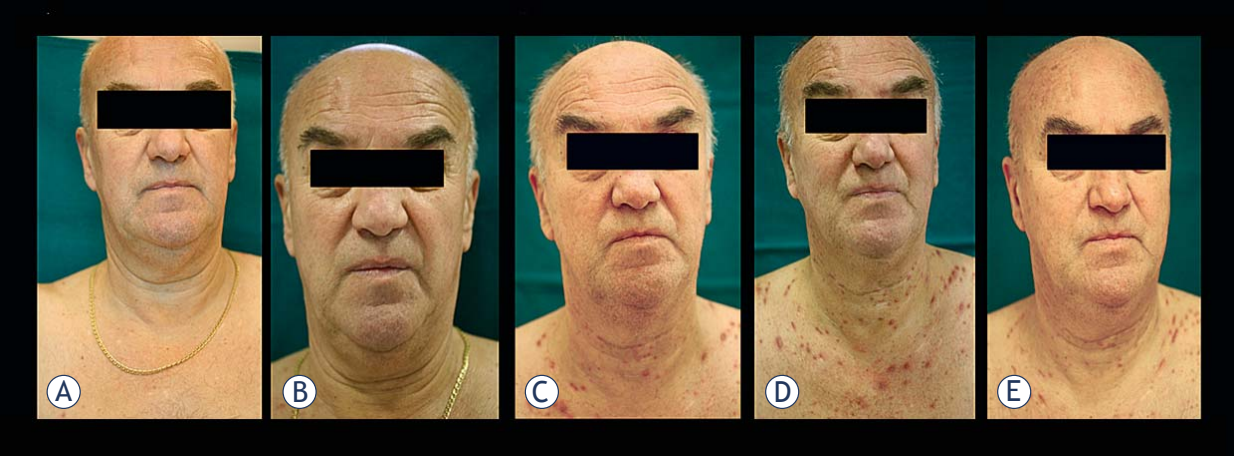
Cetuximab-related acneiform rash in a patient following prophylactic treatment with vitamin K1 cream. Vitamin K1 cream was applied to patient B twice daily from the first infusion of cetuximab and first-line chemotherapy for mCRC. Photographs are shown taken during the assessment of aceniform rash at: a) first infusion of cetuximab; b), week 1; c) week 3, d) week 4 and e) week 8

**TABLE 1.** Assessment of acenform rash in 4 patients treated with cetuximab in combination with chemotherapy and prophylactic vitamin K1 skin cream

Patient**	Weekly assessment score*								**Tumour response**

	**1**	**2**	**3**	**4**	**5**	**6**	**7**	**8**	

	**F/T**	**F/T**	**F/T**	**F//T**	**F/T**	**F/T**	**F/T**	**F/T**	
A	0/+	0/+	0/+	0/+	0/+	0/+	0/+	0/+	SD
B	0/+	0/+++	0/++	0/++	0/++	0/++	0/+	0/+	CR
C	0/++	+/+++	+/+++	0/++	0/++	0/++	0/+	0/+	PR
D	0/+	0/++	0/++	0/+	0/+	0/+	0/+	0/+	PR

* Scoring system 0=no rash; += mild rash, ++= moderate rash and +++= severe rash

** Three males and one female, average age: 61.75 years.

F= face; T= trunk; SD, stable disease; CR, complete response; PR, partial response
